# A method to screen and evaluate tissue adhesives for joint repair applications

**DOI:** 10.1186/1471-2474-13-175

**Published:** 2012-09-17

**Authors:** Tilo Dehne, Rolf Zehbe, Jan Philipp Krüger, Aneliya Petrova, Rafael Valbuena, Michael Sittinger, Helmut Schubert, Jochen Ringe

**Affiliations:** 1 Tissue Engineering Laboratory and Berlin-Brandenburg Center for Regenerative Therapies, Department of Rheumatology and Clinical Immunology, Charité-Universitätsmedizin Berlin, Föhrer Strasse 15, Berlin 13353, Germany; 2 Institute of Materials Science and Technologies, Technische Universität Berlin, Englische Strasse 20, Berlin 10587, Germany; 3 TransTissue Technologies GmbH, Charitéplatz 1, Berlin 10117, Germany; 4 Institute of Agricultural and Urban Ecological Projects, Humboldt Universität Berlin, Philippstr. 13, Berlin 10115, Germany

**Keywords:** Tissue adhesive, Bonding strength, Cartilage, Bone, Transplant, Tissue engineering, Joint repair, Test method

## Abstract

**Background:**

Tissue adhesives are useful means for various medical procedures. Since varying requirements cause that a single adhesive cannot meet all needs, bond strength testing remains one of the key applications used to screen for new products and study the influence of experimental variables. This study was conducted to develop an easy to use method to screen and evaluate tissue adhesives for tissue engineering applications.

**Method:**

Tissue grips were designed to facilitate the reproducible production of substrate tissue and adhesive strength measurements in universal testing machines. Porcine femoral condyles were used to generate osteochondral test tissue cylinders (substrates) of different shapes. Viability of substrates was tested using PI/FDA staining. Self-bonding properties were determined to examine reusability of substrates (n = 3). Serial measurements (n = 5) in different operation modes (OM) were performed to analyze the bonding strength of tissue adhesives in bone (OM-1) and cartilage tissue either in isolation (OM-2) or under specific requirements in joint repair such as filling cartilage defects with clinical applied fibrin/PLGA-cell-transplants (OM-3) or tissues (OM-4). The efficiency of the method was determined on the basis of adhesive properties of fibrin glue for different assembly times (30 s, 60 s). Seven randomly generated collagen formulations were analyzed to examine the potential of method to identify new tissue adhesives.

**Results:**

Viability analysis of test tissue cylinders revealed vital cells (>80%) in cartilage components even 48 h post preparation. Reuse (n = 10) of test substrate did not significantly change adhesive characteristics. Adhesive strength of fibrin varied in different test settings (OM-1: 7.1 kPa, OM-2: 2.6 kPa, OM-3: 32.7 kPa, OM-4: 30.1 kPa) and was increasing with assembly time on average (2.4-fold). The screening of the different collagen formulations revealed a substance with significant higher adhesive strength on cartilage (14.8 kPa) and bone tissue (11.8 kPa) compared to fibrin and also considerable adhesive properties when filling defects with cartilage tissue (23.2 kPa).

**Conclusion:**

The method confirmed adhesive properties of fibrin and demonstrated the dependence of adhesive properties and applied settings. Furthermore the method was suitable to screen for potential adhesives and to identify a promising candidate for cartilage and bone applications. The method can offer simple, replicable and efficient evaluation of adhesive properties in *ex vivo* specimens and may be a useful supplement to existing methods in clinical relevant settings.

## Background

Tissue adhesives are very useful means in a wide variety of medical procedures such as skin and wound closure [1], vascular repair [2,3], bone piece fixation in osteosurgery [4], and fixation of biomaterials and engineered transplants in cartilage repair [5].

Several factors influence the success and efficacy of a tissue adhesive like the biocompatibility, biodegradability, cost, availability and the tissue bonding properties [6]. Standardized methods to determine the strength properties of tissue adhesives are made available by standard bodies like the American Society of Testing and Materials (ASTM). These methods provide means for comparison of adhesive strength of tissue adhesives in tension, and in lap-shear or t-peel by tension loading (ASTM test methods: F2255, F2256, F2258). The common and simple structure of these experimental setups covers a broad range of medical bonding problems useful for test substrates from various tissues. Unfortunately, such test conditions often do not sufficiently reflect the biological complexity and the variety of the individual application resulting in a limited predictive power for *in vivo* testing. Therefore, the development of test methods still remains a key aspect in searching for new tissue adhesives.

In cartilage tissue engineering (TE) the fixation of tissues and transplants in the knee cavity represents a particular challenge, due to the complex mechanical loading condition. Fibrin glue has been widely-used [5], but it still has drawbacks like the danger to spread diseases [7] and cause allergenic reactions [8], and, particularly, a non-sufficient adhesive strength [9,10]. Hence there is still a high demand for alternative products.

Apart from test methods relating to ASTM, several test methods have been developed to determine adhesive strength in joint tissues. Reindel et al. developed a method to analyze the integrative repair and the adhesive strength at glued cartilage-cartilage interfaces based on thin cartilage stripes [11]. Jürgensen et al. chose for the same purpose osteochondral cylinders [12]. Hunter et al. developed an easy to handle *in vitro* cartilage repair model to analyze the integration and maturation of transplants [13]. Sierra et al. determined the failure characteristics of multiple-component adhesives [14]. Since these studies mainly focus on bonding properties of newly developed adhesive materials rather than the method itself, the description is often not sufficient to adequately reproduce. The employed equipment is customarily very complex and costly and demands a high level of technical experience and practice.

On the basis of previously published methods and existing standards we developed a method to screen and evaluate tissue adhesives intended for fixating tissues and transplants for joint repair applications. The method employs viable osteochondral tissue from porcine femoral condyles to capture aspects of joint repair applications such as bonding on cartilage and bone tissue, and surgical fixation of tissues and transplants in joint defects. The methodical approach involved steps like the generation of test tissues and the detailed presentation of used equipment and force measurement protocols resulting in an easy to use method for replication. The efficiency of this methodology was demonstrated for the adhesive properties of fibrin glue. A screening of different collagen formulations was performed to demonstrate the potential of the method to identify adhesive candidates for joint repair applications.

## Results

### Viability of tissue cylinders

**Figure 1:**
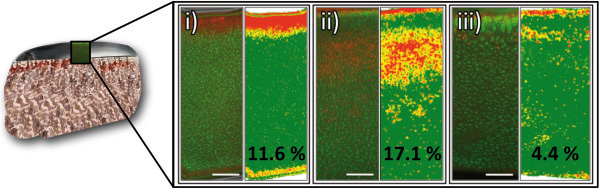
**Viability of substrate tissue. **(left) Sections of PI/FDA-stained cartilage components from (**i**) leveled and (**ii**) hole-punched substrate cylinders, and (**iii**) cartilage discs were analyzed by fluorescence microscopy. Viable cells appear green and apoptotic cells red. (right) Histomorphometric analysis was performed to determine the percentage of apoptotic cells as measure for the viability of the tissue. For each image areas of different red tones were determined and presented in new colors. High content of apoptotic cells was indicated in red, moderate in orange, low in yellow and areas with high content of viable cells were shown in green (see Methods). Only a small proportion of cells was found apoptotic (<18%), hence 48 h post preparation cartilage components of the substrates are in a viable condition for adhesive strength measurements. Bar = 200 μm.

To check whether prepared substrate tissue cylinders are still in viable condition, cell viability test with cartilage specimen was conducted 48 hours post preparation. Fluorescence microscopy of stained tissue specimen showed viable (green) and apoptotic (red) cells (Figure 1). Histomorphometric analysis of captured images was used to determine the percentage of apoptotic cells. Independent from substrate type (plane cartilage, cartilage defect cylinder or cartilage disc) more than 80% of the cells were found viable (<20% apoptotic) after 48 hours demonstrating high viability of prepared substrates for testing.

### Adhesive characteristics of fibrin glue

**Figure 2:**
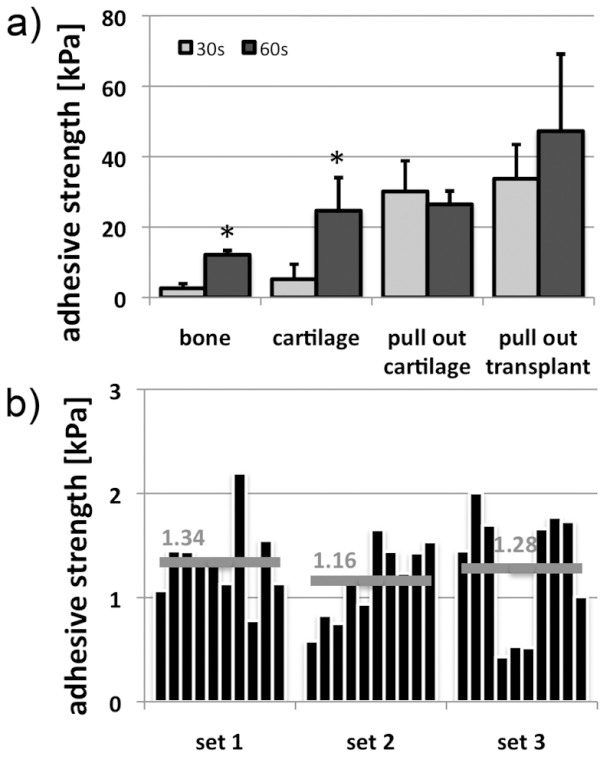
**Establishing the test system. **(**a**) Different modes of operation were applied to determine adhesive characteristics of fibrin. Varying bonding strengths were detected in the specific tests with highest values in fixing transplants and lowest in bonding bone tissue. *T*-test: 30 s vs. 60 s, * p < 0.05. (**b**) The reusability of tissue cylinders was investigated for cartilage. Three different biological replicates (set 1–3) of cartilage cylinders were investigated. After each measurement with fibrin, the adhesive was removed and a new control measurement with PBS was performed. This proceeding was repeated 10 times. Technical variations for each set range from 0.4 and 2.4 kPa, but do not increase with frequency of use suggesting that the cylinders can be reused. Differences according to biological variation are marginal (mean values range from 1.16 – 1.34 kPa, grey bar) demonstrating good reproducibility of the preparation method.

Medical grade fibrin glue was used to test the different operation modes at different assembly times (30 s, 60 s). Except for the fixation of the cartilage disc in the defect (pull-out cartilage, Figure 2a) the adhesive strength of fibrin glue was increasing with assembly time.

Varying adhesive strengths were detected in the specific tests. Highest values were obtained in pull-out test systems. Inserted cartilage was fixed by fibrin with an adhesive strength of nearly 30.1 kPa after 30 s assembly time (60 s: 26.5 kPa; -1.1-fold vs. 30 s), fibrin/PGLA-cell-transplant resisted up to 33.7 kPa after 30 s and about 47.2 kPa in average after 60 s (1.4-fold vs. 30 s). After 30 s bonding time, adhesive strength between cartilage surfaces (5.2 kPa) was significantly lower than after 60 s (24.6 kPa; 4.9-fold). In bone tissue lowest adhesive strength of all four applied systems was detected after 30 s (2.6 kPa), which was significantly increased after 60 s (12.1 kPa, 4.7-fold). For all measurements a mixture of adhesion and cohesion failure was observed. In total, adhesive strength of fibrin was increasing with assembly time on average of all operation modes (2.4-fold). Substrate failures have not occurred.

Reusability of substrates was investigated with three sets of cartilage cylinders. During repeated use no significant differences were observed compared to freshly prepared cylinders (p = 0.683, Figure 2b).

### Screening for potential tissue adhesives

The test system was used to identify potential tissue adhesives. Since collagen represents a promising material due to its biocompatibility and capability to form a natural bonding to the surrounding tissue, the screening was conducted with seven different formulations and recipes of collagen preparations from porcine skin. The assembly time was kept short (30 s) since preliminary experiments have shown that adhesive strength of collagens did not change with time (<1.1-fold vs. 60 s and 0 s).

**Figure 3:**
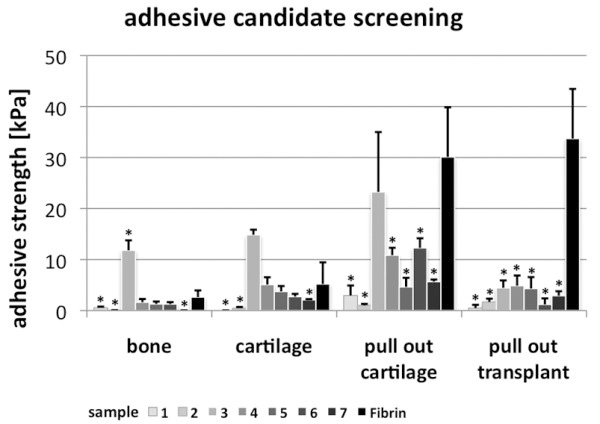
**Screening for potential adhesives. **Screening was performed in different operation modes (see Figure 3). Sample 3 demonstrated higher bonding strength than fibrin when used on cartilage and bone tissue. In settings filling “artificial defects” with cartilage tissue or TE transplants, fibrin showed highest bonding strength. These results demonstrate high bonding properties of fibrin in all settings. Sample 3 can be identified as potential tissue adhesive that is not suitable for fixing fibrin-based TE transplants. *T*-test vs. fibrin, * p < 0.05.

Collagen sample 3 demonstrated significant higher adhesive properties in cartilage (14.8 kPa) and bone tissue (11.8 kPa) compared to fibrin glue (2.6 kPa; 5.2 kPa, Figure 3). In the model filling defects with cartilage (pull-out cartilage) the detected adhesive strength of sample 3 (23.2 kPa) was almost comparable with fibrin (30.1 kPa, Figure 3). In the pull-out transplant assembly no collagen formulation was competitive with the adhesive properties of fibrin (Figure 3).

## Discussion

In an effort to quantify the adhesive bonding strength of potential medical adhesives, it is important to develop consistent and reproducible test methods for evaluation and comparative purposes. Since adhesives will be used on living tissue, a readily available test system is preferred. Available methods range from highly standardized methods with less consideration of biological aspects to tests on animal models reflecting the full complexity of a surgical problem [15].

Based on previously published methods [11-13] and on our knowledge with engineered tissues, we designed a test method providing the means to determine adhesiveness in bone and cartilage tissue. The complexity and individuality of differing applications imply that the results of a single test are not suitable for determining adhesive properties without thorough analysis and understanding of the application and adhesive behaviors. Therefore, test conditions have been developed considering surgical procedures such as the fixation of tissue and tissue engineered transplants in cartilage. Thus, not only the strength properties of the tissue adhesive at the bonding layer are considered, but also those of adherends and the behavior at the interface [16]. For bonding problems involving different tissues like bone and cartilage in joint repair, strength measurements with bone and cartilage substrates in isolation facilitate the identification of weaknesses and strength of adhesives, and hence, can contribute to improvements of the bonding material.

To test the bonding properties of tissue adhesives under *in vivo*-like conditions, vital porcine osteochondral tissue cylinders were used. Viability of the tested tissue was proven 48 h post preparation demonstrating suitability of the fabrication process for the generation of substrate tissue comprising vital cells. Tissues from bovine, ovine and porcine cylinders are preferred due to the easy availability and the fact, that relatively large samples can be harvested from a single source. The application of standard shape tools such as hole punches and tissue holders ensured the fabrication of substrate tissues with reproducible shape. For fibrin testing at cartilage surfaces the possible reusability of the prepared tissue was demonstrated. Although not meeting the high standards of material testing, the use of non-homogeneous (biological) material can be beneficial with regard to interactions between adhesive and tissue.

The efficiency of the method was shown on the basis of adhesive properties of fibrin glue. The varying results in the different test modes demonstrate that adhesives pose specific bonding properties closely related to the geometry of the applied setting. Both pull-out settings, providing a cavity filled with tissue or transplant, seem to be advantageous for fibrin, whereas blunt ends of the tissue on tissue settings are apparently not appropriate to exploit the adhesive potential of fibrin. Although difficult to compare with other studies, Sierra et al. determined similar bonding properties (around 30 kPa) for commercially available fibrin glue [14] confirming results obtained from pull-out settings. In accordance with the time dependent polymerization of fibrinogen [17], the test method was able to identify differences in adhesive strength after 30 s and 60 s assembly time. Since the collagen formulations have shown higher adhesiveness in preliminary experiments and to limit the experimental effort, screening experiments were performed only for the short time period.

We observed a mixture of adhesion and cohesion failure when fibrin was tested. Adhesive and cohesive strength determine the bonding effectiveness and can give information about adhesive properties. Ideally, cohesive failure occurs through the insufficient internal strength of the adhesive. Breaks can indicate brittleness and deformations are characteristic for e.g. polymeric elastics. Adhesion failure occurs if mechanical and intermolecular forces at the bonding layer do not resist the load [16]. The type of failure is determined by the lower strength. Combinations of both types can indicate inhomogeneities within the adhesive material or at the bonding layer. The preparation of fibrin can easily lead to inhomogeneities since fibrinogen and thrombin have to be mixed before use by the operator. Furthermore, irregularities at the substrate surface cannot be excluded completely since biological materials naturally vary.

A screening of different collagen formulations was performed to demonstrate the potential of the method to identify a promising candidate. As comparative standard fibrin glue was used, that is widely-used as adhesive in surgery [5]. The results confirm the high bonding strength of fibrin in tests filling defects such as fixing transplants or tissues. Different collagen formulations were generated using different extraction methods (urea, pepsin) and downstream modifications (oxidation, concentration, mixture with hyaluronic acid sodium salt (HA)). A test specimen extracted with pepsin and mixed with HA (sample 3) demonstrated potent adhesive properties in cartilage and bone tissue, but was not suitable for fixing fibrin-based TE transplants in defects. Other specimen extracted with pepsin (sample 5–7) or mixed with HA showed no increase of adhesive strength suggesting the formation of a unique complex between both substances. Furthermore, the varying results in the different test assemblies demonstrate that adhesive properties are closely connected with the requirements, thus, a test method has to be adapted as best to the condition of the intended application.

Generally, the method can be easily changed to torsion and shear measurements applying another force measurements device capable of it. The method was kept simple and all used equipment can easily be obtained from local providers and collaborators. The preparation technique is easily translatable to other models with comparable dimensions of joints like horse, goat and sheep [18-20]. The use of human tissue would be favorable but will be most likely not be in line with ethical considerations. Generally, the data generated from a testing method on biological tissue may vary from that found *in vivo*, however, testing results can offer valuable information on the potential bonding capacity and the preparation of subsequent *in vivo* experiments.

## Conclusion

The method represents a useful supplement to existing methods to determine adhesive strength in viable tissues in clinical relevant settings, even though not all medical procedures for cartilage or bone surgeries and TE-based interventions are covered. The method was kept simple and replicable since universal equipment was applied and detailed protocols were provided. Different test modes confirmed the dependence of bonding properties and applied settings further justifying individual test methods in addition to standard testing. Finally, the presented method was suitable to screen potential adhesives and to identify a promising candidate for cartilage and bone applications.

## Methods

### Custom-made equipment

**Figure 4:**
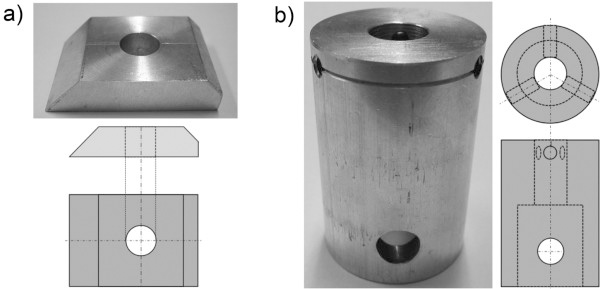
**Tissue grips. **Pictures and technical drawings of grips used for (**a**) substrate tissue cylinder preparations and (**b**) adhesive strength measurements.

Tissue grips for substrate tissues were made of aluminum or stainless steel. The grip for the preparation of tissue cylinders was designed to fit with its external dimensions to the jig of a standard microtome device. The inner bore diameter (10 mm) is matching the diameter of a tissue cylinder ensuring plane cutting of cylinder ends (Figure 4a).

The grip for force measurements comprises a cylindrical hole to hold the completed test tissue cylinders. Since tensile and compressive forces do appear during measurements, additional screws ensure adherence to exact position. The other site of the holder was designed to fit into the jig of a force measurement device (Figure 4b).

### Preparation of test substrate cylinders

**Figure 5:**
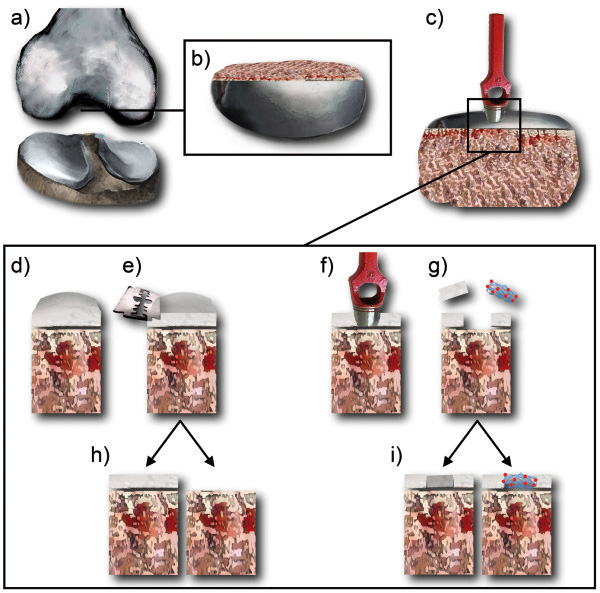
**Preparation of substrate tissues. **Schematic graphs and pictures illustrating the preparation of substrate tissue cylinders used for the determination of bonding strength. Osteochondral discs of 1–1.5 cm thickness (**b**) were obtained from the lateral and medial condyle of pig femoral bones (**a**) using a rotary reciprocating saw. (**c**) The removed discs were hole-punched to generate a cylindrical tissue specimen with a diameter of 10 mm. A bench vise facilitated a controlled agitation of the punch orthogonal to the sectional plane. (**d**) The raw tissue cylinder was placed in a customized grip that is fitting into the jig of a standard microtome device (see also Figure 4a). (**e**) Using the blade of the microtome the surface of the tissue cylinder was leveled to obtain regular bone or cartilage cylinder ends. Thus, starting from a raw cylinder (**d**), cartilage and bone substrate cylinders with plane surfaces can be generated (**h**). Furthermore, with the help of a smaller punch (ø 6 mm) another hole was created (**f**) providing an artificial cartilage defect to be filled with cartilage tissues or transplants (**g**, **i**).

To test the bonding properties of tissue adhesives in environments reflecting situations in joint repair, bone and cartilage tissue cylinders are used. Therefore, femur bones from 6–10 month-old porcine donors (100–130 kg) were purchased from a local slaughterhouse (Figure 5a). During transport to the laboratory (<3 hours) the condyle was kept covered with the meniscus preventing the cartilage from drying. For further processing, covering tissues were removed and bare condyles were cut off using a rotary reciprocating saw (Oscillant E, Aesculpap, Germany, Tuttlingen). The removed discs were 1–1.5 cm thick and 3–5 cm in diameter depending on the shape and the size of the condyle (Figure 5b). The discs were used for the preparation of the raw tissue cylinders. Accordingly, a 10 mm hole punch (Connex®, Conmetall, Germany, Celle) was used and agitated using a bench vice (Heuer, Germany, Plettenberg) to apply a controlled load force orthogonally to the sectional plane (Figure 5c). To obtain even ends tissue cylinders were cut using a custom-made tissue grip (as described above) exactly fitting into the jig of a rotary microtome device (Leica Microsystems, Germany, Wetzlar) (Figure 5e).

Finally, using raw tissue cylinders as a basis (Figure 5d), it was possible to create plane cartilage and bone tissue cylinders useful to mimic the fixation of these tissues (Figure 5h). Additional cut-outs on raw cylinders with a hole punch (Ø 6 mm Aesculap, Germany, Tuttlingen) were done to generate defined artificial defects (Figure 5f). For adhesive testing, these defects are either filled with cartilage tissue discs obtained from the previous cut-out step or with TE transplants described below (Figure 5g, h). Completed tissue cylinders were maintained until use in chondrocyte culture medium described below.

### Test of viability

Test substrate cylinders were examined for tissue viability on 0.5 mm cartilage slices involving propidium iodide/fluorescein diacetate staining (PI/FDA; Sigma-Aldrich, Germany, Taufkirchen) before use in adhesive strength testing (48 hours post preparation). Washing steps were done using PBS (Biochrom). The staining was performed first in FDA solution (3 μg/ml; 15 min, 37°C) and subsequently in PI staining solution (100 μg/ml; 2 min; RT). For microscopy an Olympus CKX41 combined with a reflected fluorescence microscopy system was used (Olympus, Hamburg, Germany). A histomorphometric analysis was performed to determine the percentage of apoptotic cells as a measure for the viability of the tissue. For each PI/FDA image the percentage of red stained areas (pixel) was determined in red-green-blue (RGB) color mode using a programming software (Xcode, Apple Inc.). Since areas containing viable and apoptotic cells appeared as mixed colors, different red tones were discriminated. Areas with high red content (red value × 0.8 > G value + B value) containing exclusively apoptotic cells were weighted as 100% apoptotic. Areas with moderate content (R × 1.0 > R + B) were weighted 50% and with low red content (R × 1.2 > R + B) 25%. Areas with other colors (R × 1.2 < = R + B) were considered as viable. After RGB analysis an image was created visualizing the discriminated areas in different colors (high – red; moderate – orange; low – yellow, other – green).

### Preparation of TE transplants

Transplants were generated adhering to protocols already published and clinically applied for matrix associated chondrocyte implantations [21]. Briefly, chondrocytes were enzymatically isolated from porcine femoral condyle cartilage. Cells were sub-cultured in medium (DMEM, Biochrom, Germany Berlin) containing 10% FBS (Biochrom). For storage cells were frozen in 10% DMSO supplemented with 80% serum and 10% medium, and were kept in liquid nitrogen until use. For transplant generation cells were thawed, sub-cultured for two weeks, and resuspended in media containing fibrinogen (33% v/v, Tissucol Duo S, Baxter, Germany, Unterschliessheim). The cell suspension was loaded and incorporated into bioresorbable co-polymer fleeces of poly(lactic-co-glycolic acid) (PLGA, 6 mm diameter, Ethicon, Germany). Addition of thrombin (10% v/v in PBS, Tissucol Duo S) led to polymerization to fibrin resulting in a ready-to-use fibrin/PLGA-cell-transplant.

### Determination of the adhesive strength

To determine the adhesive strength, a tensile test was performed. The strength of the bonding was determined using a Zwick Z005 universal testing machine (Zwick GmbH, KG, Germany, Ulm) equipped with 50 N load cell (AST GmhH, Germany, Dresden). The adhesive assembly (test substrate + adhesive) was held at 0.5 N contact load at room temperature for different contact times. Jointing components were merged with a speed of 10 mm/min until contact load was reached. After reaching assembly time, jointing components were pulled apart until rupture at a nominal speed of 5 mm/min while recording load and time. Equal volumes of fibrinogen and thrombin (20 μl each, Baxter) were applied separately on substrate tissue surfaces immediately before starting the measurement sequence. For candidate screening 20 mg material were used. For fibrin and candidate testing at least 5 repetitions were performed with freshly prepared adhesive specimen and substrates. To test the reusability of tissue substrates, three cartilage cylinder sets were used, which were reused up to 10 times.

### Modes of operation

**Figure 6:**
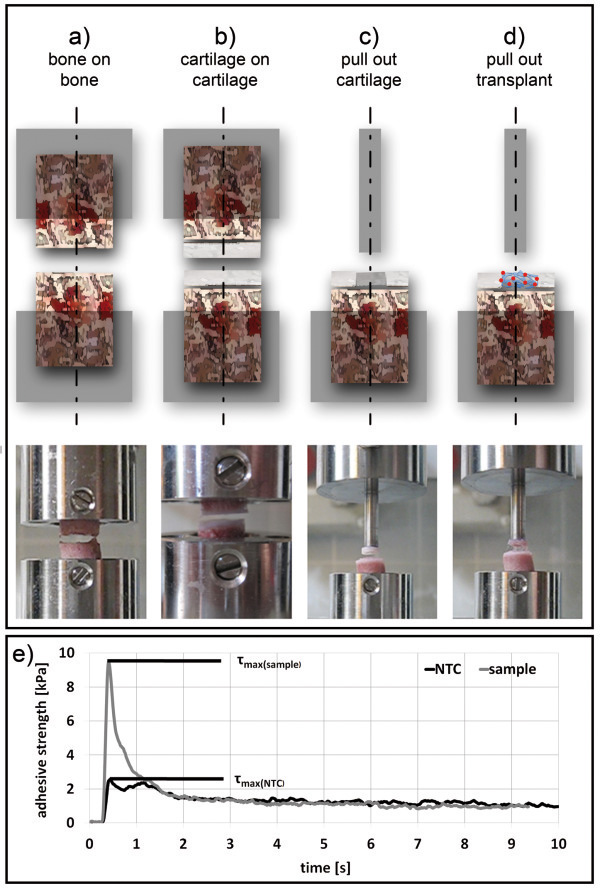
**Modes of operation. **For measuring adhesive strength in bone (**a**) or cartilage tissue (**b**), two cylinders were placed opposite to each other. For tissues and transplants a cylindrical indenter was placed opposing the inserted tissue (**c**) or transplant (**d**). The tip was coated with cyanoacrylate glue to facilitate a strong bonding between steel tip and inserted tissue or transplant after contact. After preparing the surfaces with the test adhesive, opposite sites were joined under defined initial load (e.g. 0.5 N). Once bonding time was reached opposite sites were separated with constant speed until rupture. This pulled the inserted tissue or transplants out of the “artificial defect”. (**e**) The progress of adhesive strength was recorded over time. The maximum bonding strength (τ_max_) was determined and the mean value of the base line signal was subtracted. The bonding strength was calculated from difference between “no template control” (NTC) and sample value.

For the determination of bonding strength, different modes were applied reflecting specific requirements in joint repair. For bone on bone (Figure 6a) and cartilage on cartilage (Figure 6b) settings, cylinders were placed opposite to each other and the exclusive adhesiveness on separate tissues was determined. For simulating the filling of cartilage defects, pull-out experiments for cartilage tissue (Figure 6c) and fibrin/PLGA-cell-transplants (Figure 6d) were performed. A cylindrical steel tip (custom-made, diameter 5 mm) was used as indenter and was placed opposite the inserted tissue or transplant. The indenter tip was coated with cyanoacrylate glue (Histoacryl®, Braun, Germany, Melsungen) to immediately connect indenter and sample after contact.

The bonding strength was calculated from maximum bonding strength (τ_max_) subtracted with “no template control” (NTC) bonding strength determined with phosphate buffered saline (PBS, Biochrom, Germany, Berlin). This correction was performed for each series of measurement (Figure 1), since substrate tissues demonstrated low self-adhesive properties.

### Preparation of collagen formulations

For screening tests different collagen formulations were randomly generated. Therefore, brine-conserved porcine skin was separated from fat by acetone extraction and concomitant proteins were removed through extraction with a solution containing sodium chloride (6% w/v) and formic acid (1% w/v). Collagen extraction was achieved either through extraction with 6M urea (samples 1, 2 and 4), or through exposure to 5% pepsin (samples 3, 5–7). Except sample 3 pepsin-extracted specimens were further oxidized using sodium periodate (sample 5–7). Samples 6 and 7 were additionally crosslinked with adipic acid dihydrazide. Sample 7 was concentrated by ultrafiltration before crosslinking. Sample 2 and 3 were mixed with same volume of 0.2% hyaluronic acid sodium salt (CPN spol. s r.o., Czech Republic, Dolní Dobrouc).

### Statistical analysis

Significance level of pair wise group comparison has been obtained applying *t*-test statistics of the SigmaStat 3.5 software package (Statcon, Germany, Witzenhausen). If normality or equal variance test was not passed Wilcoxon rank sum-test was conducted. A *p*-value lower than 0.05 was considered significant.

## Abbreviations

ASTM: American society of testing and materials; DMEM: Dulbecco’s modified Eagle's medium; FDA: Fluorescein diacetate; HA: Hyaluronic acid; NTC: No template control; PBS: Phosphate buffered saline; PI: Propidium iodide; PLGA: Poly(lactic-co-glycolic acid); TE: Tissue engineering.

## Competing interests

MS works as a consultant for BioTissue Technologies GmbH (Freiburg, Germany). This company develops autologous tissue transplants for the regeneration of bone and cartilage. He is also shareholder of CellServe GmbH (Berlin, Germany) and BioRetis GmbH (Berlin, Germany). The product activities of both companies have no connection with the topics discussed here. JPK works for TransTissue Technologies GmbH. TransTissue Technologies works in the field of cell-based bone and cartilage repair. All authors disclose any financial and personal relationship with other people or organizations that could inappropriately influence this scientifically oriented study.

## Author’s contribution

TD carried out adhesive strength measurements, assembly of data, data analysis and interpretation, and writing the manuscript. RZ contributed to the collection and interpretation of data, and writing the manuscript. JPK participated in the design and fabrication of custom-made devices and contributed to the development of measurement protocols. AP and RV provided the study material, contributed to the data analysis and interpretation, and participated in writing the manuscript. MS and HS participated in the conception and design of the study. JR conceived of the study, and participated in its design and coordination and helped to draft the manuscript. All authors read and approved the final manuscript.

## Pre-publication history

The pre-publication history for this paper can be accessed here:

http://www.biomedcentral.com/1471-2474/13/175/prepub
